# Safety, tolerability, and pharmacokinetics of single and multiple ascending doses of THN391, a monoclonal antibody targeting the fibrin inflammatory epitope: Phase 1a clinical trial results

**DOI:** 10.1002/alz70859_101350

**Published:** 2025-12-25

**Authors:** Aaron B Kantor, Tanja A. M. Hoffman, Dana Mannick, Ton Lisman, Jade Huguet, Elliot Offman, Rachel Schindler, Bradford Navia

**Affiliations:** ^1^ Therini Bio, Sacramento, CA USA; ^2^ SciLucent LLC, Herndon, VA USA; ^3^ University of Groningen, Groningen, Groningen Netherlands; ^4^ Certara, Montreal, QC Canada; ^5^ Schindler Neuroscience Consulting Group, New York, NY USA

## Abstract

**Background:**

Currently approved treatments for AD do not target inflammation, an essential driver of disease. These treatments are modestly effective, and present safety risks, especially for patients who are ApoE4 homozygotes. THN391 is a first‐in‐class high‐affinity humanized monoclonal antibody, targeting the inflammatory epitope on fibrin to treat inflammatory neurodegenerative diseases. At sites of vascular damage, conversion of the blood coagulation protein fibrinogen to fibrin exposes a cryptic inflammatory epitope, γ377–395, which can bind complement receptors CD11b/c on microglia, macrophages, and dendritic cells, triggering an inflammatory response. Anti‐fibrin γ377–395 antibodies significantly reduce inflammation and neuronal damage in 5XFAD mice (Ryu et al 2018).

**Method:**

THN391 was evaluated in a randomized, double‐blind, placebo‐controlled trial to assess the safety, tolerability, and PK of single and multiple ascending doses (SAD and MAD) in healthy subjects. The SAD portion comprised 6 cohorts with 8 participants each (n=6 THN391 and n=2 placebo) receiving IV doses ranging from 0.3 to 40.0 mg/kg. MAD participants received 3 doses, initially Q2W at 3.0 and 10 mg/kg, then Q4W at 20 and 40 mg/kg. Safety assessments included rotational thromboelastometry (ROTEM) to evaluate any impact on coagulation and fibrinolysis.

**Result:**

THN391 was safe and well‐tolerated at all doses with only 6 adverse events which were mild, related to the infusion site and resolved without sequelae. There were no clinically significant changes observed in lab results, vital signs, or ECG. THN391 had no impact on coagulation and fibrinolysis, as measured by PT, aPTT and ROTEM. Interim population PK modeling suggests that THN391 exhibits dose proportional pharmacokinetics and a terminal half‐life of 38 days, supporting monthly or less frequent dosing

**Conclusion:**

THN391, a first‐in‐class antibody targeting fibrin‐induced inflammation, was found to be safe and well‐tolerated in a Phase 1a study, with a half‐life supporting monthly IV dosing. A Phase 1b study is planned in patients with early AD and confirmed cerebral small vessel disease (cSVD). ApoE4 homozygotes will be included. The study will assess safety, PK, early signals of efficacy based on CSF and plasma biomarkers of inflammation and disease progression, brain MRI (ASL and DCE‐MRI) and cognition

